# Bias Calibration for Semi-Supervised Continual Learning

**DOI:** 10.3390/s26082366

**Published:** 2026-04-11

**Authors:** Zhong Ji, Zhanyu Jiao, Deyu Miao, Chen Tang

**Affiliations:** School of Electrical and Information Engineering, Tianjin University, Tianjin 300072, China; jizhong@tju.edu.cn (Z.J.); jiaozhanyu@tju.edu.cn (Z.J.); 2025439096@tju.edu.cn (D.M.)

**Keywords:** continual learning, contrastive learning, semi-supervised learning, image classification

## Abstract

In sensor-centric fields like healthcare, environmental monitoring, and industry, image classification is key to turning visual sensor data into actionable insights. Sensor-generated dynamic streaming data poses significant challenges for traditional static image classification models due to the continuous emergence of new categories, distribution shifts, and limited edge storage. With sensor streaming data facing label scarcity and high annotation costs, semi-supervised continual learning is essential, leveraging unlabeled data for incremental learning and reducing reliance on costly annotations. However, current semi-supervised continual learning methods rely on labeled data to generate pseudo-labels, leading to confirmation and relational biases. To mitigate these dual biases, we propose a Bias Calibration method based on nearest-neighbor semi-supervised continual learning, which integrates and adapts Confidence-Enhanced Learning (originally introduced for static datasets) and Guided Contrastive Learning. Specifically, the Confidence-Enhanced Learning aims to reduce competition among similar classes and penalizes low-confidence predictions, thereby generating high-confidence pseudo-labels for unlabeled data and mitigating confirmation bias. Guided Contrastive Learning constructs a pseudo-label graph and a feature representation graph, using the pseudo-label graph to optimize the feature representation graph, thereby improving class discrimination and reducing feature bias. Experiments on CIFAR-10, CIFAR-100, and ImageNet-100 show that our method significantly outperforms existing approaches, enhancing classification performance with partial labeling.

## 1. Introduction

In sensor-driven domains—encompassing healthcare monitoring, environmental surveillance, industrial inspection, and smart city sensing—image classification is pivotal for translating visual sensor data into actionable insights, such as detecting anomalies in medical imaging sensors or identifying structural defects in industrial visual sensors [1]. However, sensor environments inherently produce continuous, dynamic data streams that defy traditional static learning paradigms. Unlike fixed datasets, sensor images evolve with emerging categories (e.g., novel pollutants, unforeseen equipment malfunctions), distribution shifts (due to lighting changes, sensor drift, or seasonal variations), and limited storage for historical data on edge devices. Traditional models suffer from catastrophic forgetting when exposed to new data or become computationally prohibitive when retrained from scratch, which conflicts with sensor systems’ real-time, resource-constrained demands. Continual learning (CL) in image classification is thus indispensable: it enables models to incrementally learn new concepts from streaming sensor data while preserving prior knowledge, ensuring long-term robustness against distribution shifts and emerging categories. Without CL, sensor-based image classification would fail to adapt to dynamic real-world conditions, undermining the reliability of critical decision-making in safety-sensitive and resource-limited sensor applications.

## 2. Related Works

In real-world sensor-centric applications, data is continuously generated in dynamic streaming formats, which exhibit high volatility and frequent environmental shifts [2,3]. Unlike well-curated static datasets, sensor data streams frequently encounter emerging new categories, unpredictable distribution drifts, and variable noise levels over time. Consequently, Continual Learning (CL) becomes indispensable for sensor systems, enabling models to adapt to novel scenarios incrementally without suffering from catastrophic forgetting. Recent studies have emphasized the need for CL across diverse sensor domains. For instance, Sah et al. [2] investigated continual learning on wearable sensor data for human activity recognition, addressing the severe catastrophic forgetting problem when new sensor data arrives sequentially. Similarly, Wang et al. [3] highlighted the importance of CL in industrial equipment digital twins, proposing a method that autonomously updates models using real-time sensor data to adapt to dynamic real-world behaviors.

Despite significant progress in the field of continual learning in recent years, most advanced methods [4,5] rely on the strong assumption that data is fully labeled. This contradicts many real-world applications, as collecting labeled data is both cumbersome and expensive, especially when data is generated in a streaming format that requires real-time processing. A natural solution is to leverage semi-supervised learning methods, where data samples are not fully labeled, which has spurred research into the emerging field of semi-supervised continual learning. In continual learning, methods based on experience replay [5,6] have achieved promising results. Drawing on this idea, Kang et al. [7] adopted an experience replay paradigm in semi-supervised continual learning, using a buffer to store labeled samples and utilizing these samples to label unlabeled data in subsequent stages. However, in such methods, labeled samples are used only to label unlabeled data and are excluded from the training process, leading to two key issues: confirmation bias and feature bias.

**Figure 1:**
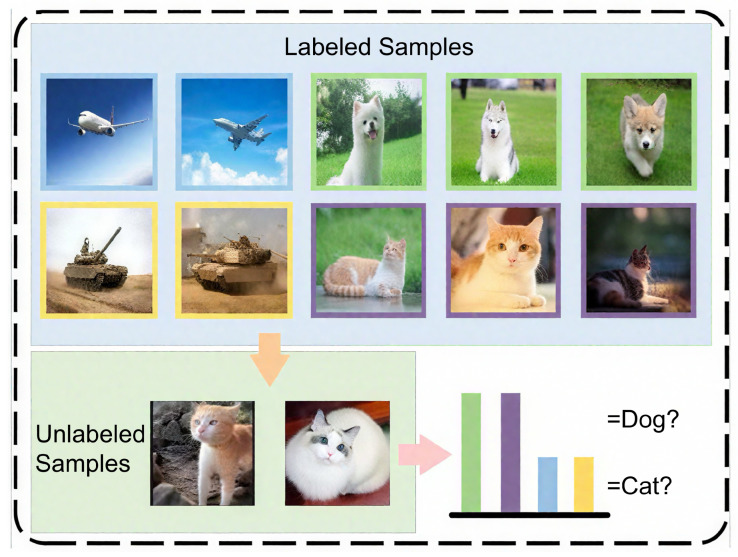
Diagram of confirmation bias. Blue, yellow, green, and purple borders denote different class groups in the support set, and the colored bars indicate class confidence scores for pseudo-label prediction.

Confirmation bias refers to the ambiguous predictions of similar classes when the model generates pseudo-labels for unlabeled samples. Since data from multiple classes is used in pseudo-label generation, pseudo-labels are susceptible to interference from different classes. When the target class is highly similar to other classes, the model makes overconfident predictions for these similar classes. As illustrated in Figure 1, when assigning pseudo-labels to unlabeled data, the relied-upon labeled data includes both current task classes (“cat” and “dog”) and previous task classes (“tank” and “airplane”). Due to the high similarity between “cat” and “dog”, the model overpredicts the “dog” class when assigning pseudo-labels to “cat”. This competition between similar classes prevents the model from generating high-confidence predictions, leading to the accumulation of bias during continuous training and exacerbating catastrophic forgetting. To mitigate this issue, we introduce and adapt Confidence-Enhanced Learning (CEL) [8] to generate high-confidence pseudo-labels for unlabeled data. Originally designed for static semi-supervised learning, CEL consists of Entropy Meaning Loss (EML) and Adaptive Negative Learning (ANL). We demonstrate that incorporating these mechanisms is highly effective at mitigating confirmation bias accumulation during continuous training on dynamic sensor streams. Specifically, EML incorporates the prediction distributions of other classes into the optimization objective to avoid competition with the target class, thereby obtaining high-confidence pseudo-labels. Meanwhile, ANL introduces additional negative pseudo-labels for all unlabeled data to utilize low-confidence samples.

Feature bias refers to the lack of discriminability in the feature representations learned by the feature extractor, which limits its ability to effectively distinguish between classes from new and old tasks during continual learning. Particularly in semi-supervised continual learning settings, only labeled data samples are replayed, leading to catastrophic forgetting of feature representations for unlabeled samples. Contrastive learning is an effective solution to this problem and has gained widespread attention in self-supervised learning. However, self-supervised contrastive learning treats all other samples in the same batch as negative samples, resulting in large variance within the same class and ambiguous class boundaries [9]. Unlike self-supervised learning, pseudo-labels can be obtained through labeled samples in semi-supervised learning. Therefore, we construct contrastive learning using pseudo-label information. According to contrastive learning theory [9], the way label information is integrated is crucial, and building an accurate pseudo-label graph to guide contrastive learning is essential. To this end, we incorporate Guided Contrastive Learning (GCL), which constructs pseudo-label graphs and feature representation graphs to measure the similarity of samples in the label space and feature space, respectively. It uses pseudo-labels to regularize the structure of the feature representation graph through graph-based contrastive learning, where samples with similar pseudo-labels are trained to have similar feature representations, thereby improving the model’s ability to distinguish between different classes in the feature space and enhancing feature discriminability.

Semi-supervised continual learning aims to reduce reliance on labels by leveraging unlabeled data. Based on the type of unlabeled data, semi-supervised continual learning methods can be divided into two categories. One category supplements the training set with unlabeled auxiliary data, alternating between supervised classification of input data and unsupervised learning of auxiliary data. Deep Model Consolidation (DMC) proposed by Zhang et al. [10] is one of the early methods to mitigate catastrophic forgetting using unlabeled auxiliary data. This method first trains a model on the labeled set and then generates pseudo-labels on the auxiliary data, where the pseudo-labels act as a regularizer between the old and new models to reduce forgetting of previous classes. Building on this, Chen et al. [11] stored a small number of instances for each incremental class as query anchors in the buffer and used these anchors to retrieve visually similar samples from the auxiliary dataset for the replay phase. In addition, Wang et al. [12] proposed Continual Test-Time Adaptation (CoTTA), which enforces consistency regularization across multiple augmented views of the same input instance and selectively fine-tunes some network parameters during the test phase, enabling the model to adapt to different scenarios. Alex et al. [13] trained an expert network that does not retain prior knowledge, focusing on achieving optimal performance on new tasks. In the next phase, this new knowledge is combined with the previous network to mitigate forgetting, and the knowledge of the old network is used to initialize the new expert.

The other category of methods does not use auxiliary data for training but reduces the original supervised data to a small portion, interleaving supervised and unsupervised data in a fixed proportion across all training batches. CNLL proposed by Karim et al. [14] is one of the early works at the intersection of semi-supervised learning and continual learning. This method trains a basic convolutional neural network model on the labeled dataset, which is then used to generate pseudo-labels on the unlabeled dataset. Subsequently, the incremental learner is fine-tuned on the pseudo-labels to implement a self-training process. Smith et al. [15] performed knowledge distillation on unlabeled data during the replay phase. Unlike other works, they identified data irrelevant to the current task using out-of-distribution detection, which not only alleviates the distribution mismatch between labeled and unlabeled data but also reduces forgetting of previously learned task classes. Wang et al. [16] proposed Online Replay with Discriminator Consistency (ORDisCo), which uses labeled data to train a Conditional Generative Adversarial Network (GAN) to capture the underlying joint distribution of partially labeled data, thereby helping the classifier make accurate predictions. Meanwhile, the classifier also performs pseudo-label prediction on unlabeled data to improve the training of the conditional generator. Brahma et al. [17] extended the generative replay scheme of ORDisCo to a meta-learning setting and proposed Meta-Consolidated Semi-Supervised Continual Learning (MCSSL), which optimizes the hyperparameters of the conditional GAN through a Variational Autoencoder (VAE) and stores the first-order statistics of incrementally learned classes for better data replay. In addition, Luo et al. [18] proposed Pseudo Gradient Learner (PGL). The use of pseudo-labels can lead to negative optimization for the classifier, resulting in the accumulation of gradient errors and a decline in performance over time. To address this issue, this method learns from labeled data via meta-learning to predict gradients for unlabeled data, thereby maintaining consistency with knowledge learned from samples of different classes. Yu et al. [19] proposed pre-training-based co-segmentation to extract knowledge from complementary foundation models, thereby generating dense pseudo-labels. They also introduced memory-based copy-paste augmentation to improve catastrophic forgetting of old classes.

## 3. Methodology

### 3.1. Problem Definition

This paper focuses on solving the semi-supervised continual learning problem in image classification. In traditional continual learning, given a data stream $D_{1},D_{2},\ldots ,D_{n},\ldots ,D_{N}$ described by tasks 1,2,…,n,…,N, the goal is to correctly classify all previously seen categories after learning new tasks. Here, the dataset associated with task *n* is denoted as $D_{n}$. Continual learning proceeds sequentially: only the current training data $D_{n}$ is available during task *n*, and previous data is systematically discarded when switching to the next task. Under semi-supervised continual learning, since the available dataset is not fully labeled, this paper further divides $D_{n}$ into a labeled subset $L_{n}$ and an unlabeled subset $U_{n}$, where $D_{n}=L_{n}\cup U_{n}$. Typically, in semi-supervised continual learning scenarios, $|L_{n}\left|\ll \right|U_{n}|$ holds, and the ratio $|L_{n}\left|/\right|U_{n}|$ remains constant across all tasks. Consistent with other experience replay-based methods, we employ a fixed-size buffer *M* to store labeled samples from old tasks, which are then combined with current labeled data to assign pseudo-labels to the unlabeled set. Additionally, to support the continual learning setting, samples in the buffer are updated via the reservoir sampling method [5], which ensures that each sample in the dataset has an equal probability of being selected into the buffer.

### 3.2. Overall Framework

**Figure 2:**
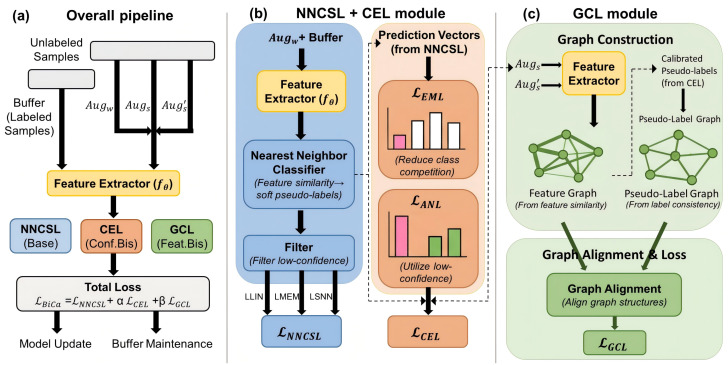
The proposed Bias Calibration (BiCa) framework. (**a**) The overall pipeline decoupling the base continual learning and bias calibration streams. (**b**) The Confidence-Enhanced Learning (CEL) module for mitigating confirmation bias. (**c**) The Guided Contrastive Learning (GCL) module for alleviating feature bias. Blue blocks denote the NNCSL stream, orange denotes CEL, and green denotes GCL; black arrows indicate data/prediction flow, and green arrows indicate graph-guided alignment optimization.

This section details the proposed Bias Calibration (BiCa) framework for semi-supervised continual learning, which consists of three components: nearest-neighbor semi-supervised continual learning, Confidence-Enhanced Learning (CEL), and Guided Contrastive Learning (GCL), as shown in Figure 2. First, we introduce nearest-neighbor semi-supervised continual learning and reveal the issues of confirmation bias and feature bias in existing semi-supervised continual learning methods. Then, in CEL, inspired by recent advances in static semi-supervised learning [8], we mitigate competition between similar classes through Entropy Meaning Loss (EML) and introduce additional negative pseudo-labels through Adaptive Negative Learning (ANL) to utilize low-confidence examples, thereby alleviating confirmation bias. Finally, we introduce GCL, which constructs pseudo-label graphs and feature representation graphs for each sample, using the pseudo-label graph to guide the optimization of the feature graph and enabling the learning of discriminative features to mitigate feature bias.

### 3.3. Nearest-Neighbor for Continual Semi-Supervised Learning

We adopt Nearest-Neighbor for Continual Semi-supervised Learning (NNCSL) [7] as the foundation. This method uses the similarity between the feature representations of labeled and unlabeled samples to provide weights for various labels of unlabeled data, and finally sums the weighted labels to generate pseudo-labels for unlabeled samples. Specifically, for an unlabeled sample, the features of two views (after strong and weak augmentations) are obtained through the model, denoted as $z_{u_{s}}$ and $z_{u_{w}}$. On this basis, the similarities between the features of labeled samples $z_{l}$ and the two features of unlabeled samples are measured respectively, and labels are aggregated according to the similarities to assign pseudo-labels to unlabeled samples. Therefore, the pseudo-label *q* under weak augmentation and the pseudo-label *p* under strong augmentation can be obtained by the following equations:

(1) $$q=\sum_{k=1}^{K}\frac{\exp\left(z_{u_{w}}\cdot z_{l}^{k}/\phi \right)}{\sum_{i=1}^{K}\exp\left(z_{u_{w}}\cdot z_{l}^{i}/\phi \right)}y^{k},\;p=\sum_{k=1}^{K}\frac{\exp\left(z_{u_{s}}\cdot z_{l}^{k}/\phi \right)}{\sum_{i=1}^{K}\exp\left(z_{u_{s}}\cdot z_{l}^{i}/\phi \right)}y^{k}$$

where *K* denotes the number of labeled samples in the current batch, and ϕ represents the temperature parameter. On this basis, the consistency of the two views is constrained: the pseudo-label obtained by strong augmentation is used as the sample label to train the network:

(2) $$L_{\mathrm{SNN}}=l\left(p,q\right)$$

where *l* denotes the cross-entropy loss function.

However, the label aggregation method inevitably causes one category to be predicted more frequently than others, leading to unbalanced or even incorrect predictions. Based on this, the NNCSL method introduces Mean Entropy Maximization (MEM) loss as a regularization term to force the distribution of all categories to remain uniform, which is defined as

(3) $$L_{\mathrm{MEM}}=h\left(\frac{1}{N}\sum_{n=1}^{N}q_{n}\right)$$

where *N* denotes the number of unlabeled samples in the current batch, and *h* denotes the entropy function.

Although the MEM loss can balance the distribution across all categories, in continual learning, the labeled and unlabeled data at the current time step come from the same distribution, whereas the historical labeled data and the current unlabeled data have different distributions. The MEM loss attempts to spread the pseudo-labels of current unlabeled data across all categories so far, which inevitably leads to incorrect pseudo-label allocation. To correct this error, the NNCSL method filters out historical labeled samples based on the labels of all previously learned categories, ensuring that filtering does not affect the pseudo-label allocation in Equation (1). Meanwhile, for all labeled data, the output of the linear classifier is used to optimize the standard cross-entropy loss:

(4) $$L_{\mathrm{LIN}}=\sum_{k=1}^{K}l\left(v^{k},y^{k}\right)$$

Here, v denotes the output of the linear classifier, and y denotes the true label of the sample. Through the three losses above, NNCSL can assign relatively accurate pseudo-labels to unlabeled samples. However, for continual learning, it is necessary to retain old knowledge while learning new knowledge. In the NNCSL method, Nearest Neighbor Distillation (NND) is introduced to maintain the consistency of pseudo-labels for the same sample across different time points via knowledge distillation, thereby achieving knowledge retention. Specifically, NNCSL denotes the model at time *t* as $f_{\theta }^{t}$, and the model at time t−1 as $f_{\theta }^{t-1}$. For each unlabeled sample, the feature outputs of the model at times t−1 and *t* are denoted as $z_{u}^{t-1}$ and $z_{u}^{t}$, respectively. By constructing the corresponding nearest-neighbor classifier with the labeled set of the corresponding time, the corresponding pseudo-labels are obtained. Thus, the pseudo-labels at times t−1 and *t* are expressed as

(5) $$w^{t-1}=\sum_{k=1}^{K}\frac{\exp\left(z_{u}^{t-1}\cdot z_{k}^{t-1}/\phi \right)}{\sum_{i=1}^{K}\exp\left(z_{u}^{t-1}\cdot z_{i}^{t-1}/\phi \right)}y^{k},\;w^{t}=\sum_{k=1}^{K}\frac{\exp\left(z_{u}^{t}\cdot z_{k}^{t}/\phi \right)}{\sum_{i=1}^{K}\exp\left(z_{u}^{t}\cdot z_{i}^{t}/\phi \right)}y^{k}$$

Here, $z_{l}^{t-1}$ and $z_{l}^{t}$ denote the labeled set features embedded in the old and new feature spaces, respectively. Thus, pseudo-labels for each unlabeled sample at different times are obtained, and knowledge retention is achieved by maintaining consistency in category relationships across time. The Nearest Neighbor Distillation can be expressed as

(6) $$L_{\mathrm{NND}}=H\left(w^{t},w^{t-1}\right)$$

Finally, the loss function of the NNCSL method can be expressed as

(7) $$L_{\mathrm{NNCSL}}=L_{\mathrm{SNN}}+\lambda_{\mathrm{MEM}}L_{\mathrm{MEM}}+\lambda_{\mathrm{NND}}L_{\mathrm{NND}}$$

### 3.4. Confidence-Enhanced Learning

The previous section briefly introduced the NNCSL method, which stores labeled samples in a buffer and uses them to label unlabeled data in the next phase. Although this method has become the cornerstone of many approaches due to its simplicity and effectiveness, during pseudo-label assignment, it uses feature distances between unlabeled and labeled data to assign weights to pseudo-labels and obtain soft pseudo-labels. When there are similar classes in the labeled data, confirmation bias will occur, thereby reducing the confidence of pseudo-labels. To mitigate this bias, this section adopts Confidence-Enhanced Learning (CEL) proposed by Chen et al. [8] to improve the confidence of pseudo-labels, thereby reducing the accumulation of confirmation bias during continual training. Specifically, this section first adopts a threshold-based strategy to select the target class and, at the same time, imposes additional supervision on the predictions of non-target classes to push them toward a uniform distribution. Specifically, this section uses $Q^{\left(i\right)}=\left[q_{1}^{\left(i\right)},\ldots ,q_{C}^{\left(i\right)}\right]$ to define the prediction vector of sample *i* generated by weak augmentation under the soft nearest neighbor classification strategy. Meanwhile, $S^{\left(i\right)}=\left[s_{1}^{\left(i\right)},\ldots ,s_{C}^{\left(i\right)}\right]\in \left\{0,1\right\}$ denotes a binary vector for label selection, where $s_{c}^{\left(i\right)}=1$ indicates that category *c* is selected as the target category, and $s_{c}^{\left(i\right)}=0$ indicates that this category lacks a specific label.

This binary vector can be calculated as

(8) $$s_{c}^{\left(i\right)}=I\left(q_{c}^{\left(i\right)}\geq \tau \right)$$

where τ is the selection threshold. In addition, we can compute the vector $U^{\left(i\right)}=\left[u_{1}^{\left(i\right)},\ldots ,u_{C}^{\left(i\right)}\right]$ for selecting non-target categories, where $u_{c}^{\left(i\right)}=1$ indicates that category *c* is a non-target category. It is expressed as:

(9) $$u_{c}^{\left(i\right)}=I\left(\max(Q^{\left(i\right)})\geq \tau \right)\cdot I\left(s_{c}^{\left(i\right)}=0\right)$$

For the prediction vector of sample *i* generated by strong augmentation, this section denotes it as $P^{\left(i\right)}=\left[p_{1}^{\left(i\right)},\ldots ,p_{tc}^{\left(i\right)},\ldots ,p_{C}^{\left(i\right)}\right]$, where $p_{tc}^{\left(i\right)}=I\left(u_{c}^{\left(i\right)}=0\right)\cdot p_{c}^{\left(i\right)}$ represents the confidence of the target category in the strong-augmented prediction. Through the optimization of the unsupervised loss function (i.e., cross-entropy), $p_{tc}^{\left(i\right)}$ will gradually converge to the given label; as the model is continuously trained, the confidence in the pseudo-label category gradually increases to 1.

However, in some challenging cases, competition between non-target and target categories always leads to the failure to generate high-confidence predictions. To address this issue, we impose an additional constraint on other categories (i.e., all non-target categories), allowing them to equally share the remaining confidence $1-p_{tc}^{\left(i\right)}$, thereby avoiding category competition with the target category. This can be expressed as

(10) $$y_{c}^{\left(i\right)}=\frac{1-I\left(u_{c}^{\left(i\right)}=0\right)\cdot p_{c}^{\left(i\right)}}{\sum_{c}I\left(u_{c}^{\left(i\right)}=1\right)}$$

where $y_{c}^{\left(i\right)}$ is the label of the non-target category. It indicates that once the prediction probability for the target category is determined, the remaining confidence score will be equally distributed among the non-target categories. It is worth noting that EML is only applied to samples assigned with pseudo-labels, which means $\sum_{c}I\left(u_{c}^{\left(i\right)}=1\right)$ is always greater than 0 (since $\max(Q^{\left(i\right)})\geq \tau$). Since $y_{c}^{\left(i\right)}\in \left[0,1\right]$, the Binary Cross-Entropy (BCE) loss can be used to train the model. Thus, following [8], the Entropy Meaning Loss (EML) is defined as:

(11) $$L_{\mathrm{EML}}=-\frac{1}{NC}\sum_{i=1}^{N}\sum_{c=1}^{C}u_{c}^{\left(i\right)}\left[y_{c}^{\left(i\right)}\log\left(p_{c}^{\left(i\right)}\right)+\left(1-y_{c}^{\left(i\right)}\right)\log\left(1-p_{c}^{\left(i\right)}\right)\right]$$

though EML alleviates the competition between categories to a certain extent, the strategy of optimizing pseudo-labels using a threshold strategy cannot utilize all unlabeled data. In more complex cases, a large number of unlabeled samples are filtered by the threshold and not assigned pseudo-labels, resulting in no contribution to model optimization. For example, the maximum confidence is only 0.3, while the threshold is 0.95. This prompts us to further consider how to utilize unlabeled examples with low confidence without pseudo-labels (i.e., with maximum confidence much lower than the predefined threshold). Intuitively, the prediction may be confused among the top-ranked classes, but it will determine that the input example does not belong to the classes ranked after these classes. To this end, we utilize Adaptive Negative Learning (ANL) [8], which assigns an additional negative pseudo-label to the determined non-target classes and uses all unlabeled samples for training, thereby better alleviating confirmation bias. Specifically, we first compute the temporary label based on the weak-augmented prediction, regardless of whether the maximum confidence exceeds the threshold. Since model performance can be reflected by the prediction consistency of different augmented inputs [20], we treat the obtained temporary label as the true label of the strong-augmented version, and calculate the minimum *k* such that its top-*k* accuracy reaches 100%. This can be expressed as

(12) $$k=\arg\min_{\theta \in [2,C]}\left(\mathrm{Acc}\left(P^{t},\hat{Q},\theta \right)=100\%\right)$$

where $\hat{Q}=\arg\max\left(Q,t\right)$ is the temporary label at step *t*, and $P^{\left(t\right)}$ is the strong-augmented prediction vector of unlabeled samples (calculated over the entire batch of samples). Acc and *C* denote the function for calculating top-*k* accuracy and the number of categories, respectively. Since there are always some examples without pseudo-labels in each training step, and we compute *k* over all unlabeled data, overfitting can be alleviated.

Finally, we assign negative pseudo-labels to categories ranked after top-*k* in the weak-augmented prediction distribution. Thus, the vector in Equation (8) can be recalculated as

(13) $$s_{c}^{\left(i\right)}=I\left(q_{c}^{\left(i\right)}\geq \tau \right)+I\left(\mathrm{Rank}(q_{c}^{\left(i\right)})>k\right)$$

Here, Rank is a category-sorting function that orders categories by descending confidence scores. In the early stages of training, when the model is fed different augmented versions of the same sample, the output distributions differ significantly—thus, the value of *k* is increased. When k=C, ANL provides no negative pseudo-labels. Through the optimization of consistency loss (i.e., cross-entropy loss), the model exhibits stronger output invariance to input noise, the value of *k* decreases, and more negative pseudo-labels are selected. This adaptive negative learning loss can be expressed as

(14) $$L_{\mathrm{ANL}}=-\frac{1}{N}\sum_{i=1}^{N}\sum_{c=1}^{C}I\left[\mathrm{Rank}(q_{c}^{\left(i\right)})>k\right]\log\left(1-p_{c}^{\left(i\right)}\right)$$

Here, ANL is a threshold-independent scheme, so it can be applied to all unlabeled data. The total loss obtained by combining Entropy Meaning Loss (EML) and Adaptive Negative Learning Loss (ANL) is as follows:

(15) $$L_{\mathrm{CEL}}=\alpha L_{\mathrm{EML}}+\left(1-\alpha \right)L_{\mathrm{ANL}}$$

where α is a hyperparameter that balances these two terms. By optimizing Equation (15), we integrate the efficient and flexible Confidence-Enhanced Learning (CEL) method [8] into our continual learning pipeline. In this way, the confirmation bias caused by similar categories during pseudo-label assignment is alleviated, thereby further mitigating the catastrophic forgetting problem.

### 3.5. Guided Contrastive Learning

In the previous subsection, we mitigated confirmation bias at the output level of the model. However, in semi-supervised continual learning, learning powerful discriminative features is also a challenging task. This is because with limited labeled data, the estimation of the feature space is more likely to be erroneous, leading to feature bias. To address this challenge, we introduce a Guided Contrastive Learning (GCL) module into our continual learning framework. The core formulation of this module, including the memory-smoothed pseudo-labeling and the graph-based contrastive regularization (Equations (16)–(22)), is inspired by and directly adapted from CoMatch [21]. While CoMatch was originally designed for standard static semi-supervised learning, we discovered that its mechanism is exceptionally effective in the Semi-Supervised Continual Learning (SSCL) domain. By constructing a pseudo-label graph and a feature representation graph identically to CoMatch [21], and using the former to guide the latter, we enforce the model to learn highly discriminative features across sequential tasks. This effectively prevents the feature extractor from losing its discriminative power over old classes, thereby successfully alleviating the severe feature bias inherent in SSCL. Next, we first introduce the process of obtaining the pseudo-label graph, followed by a detailed description of the adapted guided contrastive learning algorithm.

Previous methods utilize a relational graph in which edges represent “similarity” or “dissimilarity” between nodes. Similar nodes are forced to output consistent features, while dissimilar nodes are forced to output inconsistent features. However, since unlabeled data may be incorrectly labeled, the judgment of edges may be unreliable. To address this issue, we use the feature representations of samples to impose a smoothness constraint on class probabilities for pseudo-label refinement. The refined pseudo-labels are then used to construct the pseudo-label graph, thereby mitigating prediction bias. Specifically, for samples in the given labeled and unlabeled sets, we obtain the weakly-augmented feature representation $z^{w}$ via weak augmentation, and then derive their class probabilities. For labeled samples, the class probability is defined as their true label $p^{w}=y$; for unlabeled samples, it is defined as the model’s prediction for their weakly augmented version, as in Equation (1). In Equation (1), pseudo-labels are generated by aggregating the labels of labeled samples in the same batch, but such generated pseudo-labels ignore the connections among unlabeled samples. Therefore, we optimize the pseudo-labels by aggregating the class probabilities of unlabeled samples in the same batch. Specifically, following CoMatch [21], this is achieved by optimizing the following objective function:

(16) $$J\left(q_{b}\right)=\left(1-\beta \right)\sum_{n=1}^{N}a_{n}{∥q_{b}-p_{n}^{w}∥}_{2}^{2}+\beta {∥q_{b}-p_{b}^{w}∥}_{2}^{2}$$

Here, the first term represents a smoothness constraint: It encourages $q_{b}$ to take values similar to the category probabilities of its neighboring unlabeled samples, while the second term attempts to preserve its original category prediction. β is a hyperparameter for balancing the two terms. $a_{n}$ measures the affinity between the current sample and the *n*-th unlabeled sample in the same batch, and is calculated using the similarity in the feature space:

(17) $$a_{n}=\frac{\exp\left(z_{b}^{w}\cdot z_{n}^{w}/t\right)}{\sum_{n=1}^{N}\exp\left(z_{b}^{w}\cdot z_{n}^{w}/t\right)}$$

Here, *t* is a scalar temperature parameter. Since $a_{n}$ is normalized (i.e., the sum of $a_{n}$ equals 1), when $J(q_{b})$ is optimized to its minimum value, the pseudo-label $q_{b}$ can be derived as follows:

(18) $$q_{b}=\beta p_{b}^{w}+\left(1-\beta \right)\sum_{n=1}^{N}a_{n}p_{n}^{w}$$

For the pseudo-labels of each unlabeled sample in a given batch, the pseudo-label graph is constructed identically to [21] by building a similarity matrix $W^{q}$:

(19) $$W_{bj}^{q}=\left\{\begin{array}{cc} 1 & \mathrm{if}\;b=j\\ q_{b}\cdot q_{j} & \mathrm{if}\;b\neq j\;\mathrm{and}\;q_{b}\cdot q_{j}\geq T\\ 0 & \mathrm{otherwise} \end{array}\right.$$

Here, samples with similarity below the threshold *T* are not connected, and each sample is connected to itself with the strongest edge value of 1. The constructed pseudo-label graph serves as the target for the training feature representation graph. To construct the feature representation graph, we first apply two strong augmentations to each unlabeled sample $u_{b}$ and obtain their features:

$$z_{b}^{\prime }=g\circ f\left(Aug_{s^{\prime }}\left(u_{b}\right)\right)$$

Then, the feature representation graph $W^{z}$ is constructed as follows:

(20) $$W_{bj}^{z}=\left\{\begin{array}{cc} \exp\left(z_{b}\cdot z_{b}^{\prime }/t\right) & \mathrm{if}\;b=j\\ \exp\left(z_{b}\cdot z_{j}/t\right) & \mathrm{if}\;b\neq j \end{array}\right.$$

Our goal is to train the encoder and projection head such that the feature representation graph has the same structure as the pseudo-label graph. To this end, we first normalize $W^{q}$ and $W^{z}$ so that the sum of each row in the similarity matrix equals 1. Then we minimize the cross-entropy between the two normalized graphs. The contrastive loss is defined as [21]:

(21) $$L_{\mathrm{GCL}}=\frac{1}{N}\sum_{b=1}^{N}H\left({\hat{W}}_{b}^{q},{\hat{W}}_{b}^{z}\right)$$

It can be decomposed into two terms:

(22) $$-{\hat{W}}_{bb}^{q}\log\left(\frac{\exp(z_{b}\cdot z_{b}^{\prime }/\tau )}{\sum_{j=1}^{N}{\hat{W}}_{bj}^{z}}\right)-\sum_{\begin{array}{c} j=1\\ j\neq b \end{array}}^{N}{\hat{W}}_{bj}^{q}\log\left(\frac{\exp(z_{b}\cdot z_{j}/\tau )}{\sum_{j=1}^{N}{\hat{W}}_{bj}^{z}}\right)$$

The first term comes from the self-loop in the pseudo-label graph: it encourages the model to generate similar feature embeddings for different augmentations of the same image. The second term encourages samples with similar pseudo-labels to have similar embeddings—it clusters samples of the same category, achieving entropy minimization. By optimizing the above contrastive loss adopted from CoMatch [21], the pseudo-label graph is utilized to guide feature representation learning. We discover that this powerfully improves the model’s ability to distinguish different categories in the feature space across sequential tasks, effectively enhancing feature discriminability in the continual learning stream. Finally, the complete objective function of this section includes three components: Nearest-Neighbor Semi-Supervised Continual Learning, Confidence-Enhanced Learning, and Guided Contrastive Learning. The model is optimized by jointly constraining these three losses during the training phase. The complete objective function is expressed as:

(23) $$L_{\mathrm{BiCa}}=L_{\mathrm{NNCSL}}+\delta_{1}\cdot L_{\mathrm{CEL}}+\delta_{2}\cdot L_{\mathrm{GCL}}$$

where $\delta_{1}$ and $\delta_{2}$ are hyperparameters used to balance the effects of each loss term.

## 4. Experiments

### 4.1. Experimental Setup

#### 4.1.1. Dataset

The method proposed in this paper is evaluated on three datasets: CIFAR-10 [22], CIFAR-100 [22], and ImageNet-100 [23].

CIFAR-10 was proposed by Hinton et al. in 2009. It consists of 60,000 32×32 pixel color images covering 10 distinct categories: airplane, automobile, bird, cat, deer, dog, frog, horse, ship, and truck. To ensure model training and evaluation, each category contains 5000 training images and 1000 test images.

CIFAR-100 is an extension of CIFAR-10. Both datasets consist of 50,000 training images and 10,000 test images, with each image having a resolution of 32×32 pixels. However, there is a significant difference in their categories: CIFAR-100 includes 100 categories, which are grouped into 20 superclasses (each superclass contains 5 subclasses). For example, “bicycle” is a subclass belonging to the superclass “vehicle”. In terms of quantity, each category includes 500 training images and 100 test images.

ImageNet-100 is a 100-category subset of the ImageNet-1k dataset from the 2012 ImageNet Large Scale Visual Recognition Challenge. Each color image in this dataset has a resolution of 64×64 pixels. To ensure the accuracy of training and evaluation, each category contains 1300 training images and 50 test images.

#### 4.1.2. Experimental Details

In terms of experimental details, BiCa adopts ResNet-18 as the network backbone, with 250 training epochs across all datasets. For the CIFAR-10 and CIFAR-100 datasets, experiments are conducted with buffer sizes of 500 and 5120; for the ImageNet-100 dataset, experiments are performed only with a buffer size of 5120. In all experiments, current data and buffer data are organized in batches, with each batch size set to 256. At the end of each batch, labeled samples in the buffer are updated in the data stream.

Following the standard class-incremental learning protocol, we systematically partition the datasets into multiple disjoint sequential tasks. Specifically, the CIFAR-10 dataset is divided into 5 sequential tasks (2 distinct classes per task), and the CIFAR-100 dataset is constructed into 10 consecutive tasks (10 new classes per task). For the ImageNet-100 dataset, evaluation is performed with 20 consecutive tasks (5 classes per task). Regarding the data augmentation strategy required by our framework for pseudo-label generation and consistency regularization, we employ distinct operations. Weak augmentation consists of standard random cropping and random horizontal flipping. Strong augmentation utilizes RandAugment combined with Cutout, which applies severe geometric transformations and color distortions to generate highly perturbed views.

To rigorously justify the hyperparameter selection, we utilized a grid search strategy on a held-out validation set during the initial tasks. Specifically, the threshold parameter τ is set to 0.8, the balance hyperparameter α is set to 0.5, the contrastive threshold *T* is set to 0.8, and the temperature coefficient *t* is set to 0.07. Detailed experimental justifications and sensitivity analyses for selecting these optimal values (τ, α, and *T*) are extensively discussed and visualized in Section 4.4 (Parameter Experiments). The proposed algorithm is implemented using PyTorch v2.2.2 framework (PyTorch Foundation, San Francisco, CA, USA) and trained and tested on NVIDIA GeForce RTX 4090 GPUs and NVIDIA Tesla V100 GPUs (NVIDIA Corporation, Santa Clara, CA, USA).

### 4.2. Experimental Results

In this section, the proposed BiCa method is compared with current state-of-the-art continual learning methods. Specifically, for the CIFAR-10 and CIFAR-100 datasets, we select ten methods for comparison: ER [24], iCaRL [25], DER [6], GDumb [26], PseudoER [27], CIC [27], CCIC [27], PAWS [28], NNCLS [7], and the recent 2025 state-of-the-art method NI-SSCL [29]. Among them, CCIC [27] is a representative semi-supervised continual learning framework, while PseudoER (Experience Replay with pseudo-labels) and CIC are two of its foundational baseline variants introduced in the same work. Including these variants allows for a more comprehensive and granular comparison. For the ImageNet-100 dataset, we select seven methods: ER [24], FOSTER [30], X-DER [25], CCIC [27], PAWS [28], NNCLS [7], and NI-SSCL [29]. Note that the results for NI-SSCL [29] are directly reported from the original paper under identical experimental settings. Since the original work did not report performance for a buffer size of 5120, these specific entries are marked as ‘-’ in our comparative tables. The experimental results represent the average accuracy across five runs with different initializations. Notably, the proposed BiCa method consistently achieves highly competitive or superior performance under all settings.

Table 1 and Table 2 present the experimental results on CIFAR-10 and CIFAR-100, respectively. It can be observed that the proposed BiCa method outperforms the previous baselines in almost all cases. Specifically, on CIFAR-10, BiCa improves the accuracy by at least 0.68% compared to other methods; particularly when the buffer size is set to 500, and the label ratio is 0.8%, it outperforms NNCSL [7] (the second-highest previous baseline) by 3.36%. Additionally, BiCa also demonstrates strong advantages on CIFAR-100: taking a buffer size of 5120 as an example, BiCa achieves performance improvements of 1.1%, 2.2%, and 2.32% over the next-best prior method at label ratios of 0.8%, 5%, and 25%, respectively.

Furthermore, when rigorously benchmarked against the latest 2025 state-of-the-art method, NI-SSCL [29], our proposed BiCa framework continues to demonstrate highly robust and competitive performance. We transparently observe that while NI-SSCL marginally outperforms our method on certain specific intermediate metrics (e.g., yielding 40.01% vs. 36.62% in one specific CIFAR-100 setting), BiCa successfully surpasses NI-SSCL [29] on the majority of the final accuracy evaluations (e.g., achieving 84.11% vs. 83.23% and 58.73% vs. 57.86%). More importantly, it is crucial to interpret these highly comparable results through the lens of fundamental architectural differences. NI-SSCL heavily relies on a “Neuron Expansion” module [29], which dynamically increases the network’s capacity and structural-parameter count over time to accommodate new knowledge. In stark contrast, our BiCa framework operates under a strictly fixed, lightweight network architecture (Static Backbone). Given our target application of sensor-generated dynamic streaming data, edge devices are typically bound by rigorous memory and computational constraints, making a static architecture practically mandatory. Strikingly, even without the unfair structural advantage of dynamic network expansion, BiCa achieves superior or matching performance. This explicitly demonstrates the exceptional efficiency of our bias calibration mechanisms in maximizing the learning potential of a fixed-capacity network in edge-centric scenarios.

Furthermore, Table 1 and Table 2 reveal noticeable performance variations of BiCa across different datasets, buffer sizes, and label ratios. Specifically, BiCa achieves lower accuracy on CIFAR-100 than on CIFAR-10, which is because CIFAR-100 contains more categories and is more challenging. For the same dataset, the performance difference across different buffer sizes arises because a larger buffer can better alleviate confirmation bias and feature bias (by including more samples). Moreover, model performance gradually improves as the label ratio increases—this is because more labeled samples are used to assign pseudo-labels to unlabeled data, resulting in more robust pseudo-labels; training with these pseudo-labels thus yields higher performance.

Table 3 presents the average accuracy comparison between BiCa and other methods on the ImageNet-100 dataset. This experiment is conducted under the setting of 20 consecutive semi-supervised tasks and a buffer size of 5120. Similarly, the proposed BiCa method achieves the highest accuracy under all settings: specifically, when the proportion of labeled data is 1%, 5%, and 25%, BiCa’s accuracy reaches 30.93%, 52.74%, and 67.16%, respectively, outperforming the suboptimal NNCSL [7] method by 1.01%, 1.39%, and 1.41%. As the proportion of labeled data increases, BiCa’s advantages become more prominent: this is because more labeled samples are used to generate pseudo-labels, thereby assigning high-confidence pseudo-labels to unlabeled samples for training.

Furthermore, we observe that methods using the nearest-neighbor strategy to generate pseudo-labels (PAWS [28], NNCSL [7], and BiCa) perform far better than the other comparison methods. Specifically, when the labeled data proportion is 25%, the proposed BiCa method outperforms X-DER [25] by 21.34%. This demonstrates that using the nearest-neighbor strategy to obtain pseudo-labels provides a stronger advantage when learning high-resolution images with large variance.

### 4.3. Ablation Experiments

To evaluate the impact of different components in BiCa on model performance, this section conducts relevant ablation studies on the CIFAR-10 and CIFAR-100 datasets with a buffer size of 500. The experimental results are shown in Table 4. Since our proposed BiCa method improves upon NNCSL [7], we use NNCSL as the baseline. On this basis, we observe the influence of the nearest neighbor semi-supervised continual learning loss $L_{\mathrm{NNCSL}}$, entropy meaning loss $L_{\mathrm{EML}}$, adaptive negative learning loss $L_{\mathrm{ANL}}$, guided contrastive learning loss $L_{\mathrm{GCL}}$, and combinations of these losses on accuracy. Here, the Confidence-Enhanced Learning loss $L_{\mathrm{CEL}}$ is split into $L_{\mathrm{EML}}$ and $L_{\mathrm{ANL}}$ to better analyze the role of each component.

The results show that each loss function has a positive impact on improving model performance. We observe that although both $L_{\mathrm{EML}}$ and $L_{\mathrm{ANL}}$ improve model performance to varying degrees, when they are combined into Confidence-Enhanced Learning, the model performance is better than their individual performance. Taking CIFAR-10 as an example: when the labeled data ratio is 0.8%, Confidence-Enhanced Learning outperforms $L_{\mathrm{EML}}$ and $L_{\mathrm{ANL}}$ (used alone) by 0.69% and 0.64%, respectively. This trend also holds when $L_{\mathrm{GCL}}$ is included, which proves that $L_{\mathrm{EML}}$ and $L_{\mathrm{ANL}}$ are complementary. Compared with Confidence-Enhanced Learning, guided contrastive learning brings a more significant improvement to the baseline. For instance, on CIFAR-10 with a 0.8% labeled ratio, there is a 1.09% performance gap between the two components. This is because guided contrastive learning guides feature representation learning using the pseudo-label graph, thereby improving the model’s ability to distinguish between categories in the feature space.

Meanwhile, we can also observe that the combination of Confidence-Enhanced Learning and guided contrastive learning outperforms their individual performances. For example, on CIFAR-100 with a 0.8% labeled ratio, the combined performance of the two components is 0.58% and 0.52% higher than their individual use, respectively. This indicates that the two components are mutually reinforcing: specifically, Confidence-Enhanced Learning enables the model to generate high-confidence pseudo-labels by alleviating confirmation bias, while guided contrastive learning alleviates bias at the feature level, thereby improving feature discriminability.

### 4.4. Parameter Experiments

**Figure 3:**
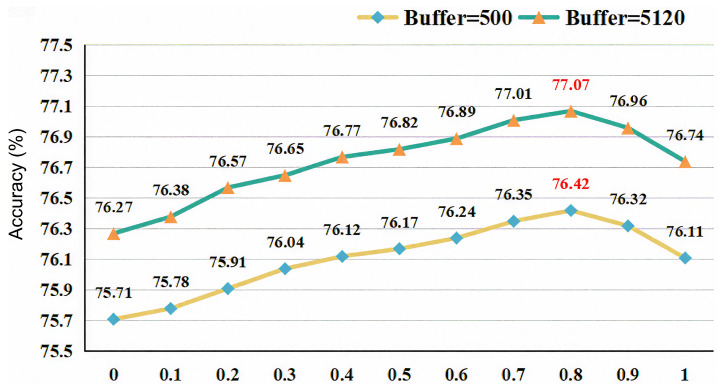
The impact of different parameter τ on the CIFAR-10 dataset. Red numbers mark the peak performance points.

This section conducts experiments and analysis on the parameters of the BiCa method. Among them, Figure 3 shows the impact of parameter τ (in Equation (8)) on model performance on the CIFAR-10 dataset. Specifically, as τ increases, the model’s performance gradually improves until it reaches a peak at τ=0.8, after which the performance gradually decreases. Additionally, we observe that the model performance in the interval [0.7, 0.9] is significantly better than in other cases. This is because τ acts as a filter for pseudo-labels to help the model generate high-confidence pseudo-labels: a too-low threshold fails to filter pseudo-labels, while a too-high threshold (since pseudo-labels are generated by the nearest neighbor classifier) filters out a large number of unlabeled samples, making no contribution to model optimization.

**Figure 4:**
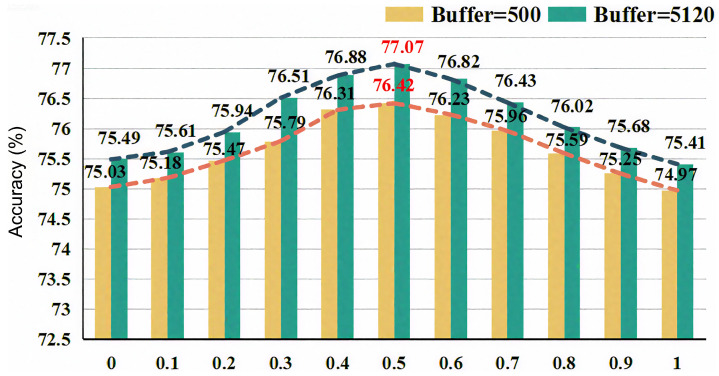
The impact of different parameter α on the CIFAR-10 dataset. Yellow and green bars represent results with buffer sizes of 500 and 5120, respectively; the orange and dark-blue dashed lines indicate the corresponding trend curves; red numbers mark the peak performance points.

Figure 4 demonstrates the impact of changes in hyperparameter α (in Equation (15)) on model performance, under the condition of a 0.8% labeled data ratio on the CIFAR-10 dataset. The experimental results show that as α increases from 0 to 1, the model performance first rises and then falls, reaching an optimal value at α=0.5. Moreover, this trend is consistent for both buffer sizes of 500 and 5120. This is because, in Confidence-Enhanced Learning, the entropy-based loss alleviates competition between similar categories by allowing non-target categories to share the remaining confidence; the adaptive negative learning introduces additional negative pseudo-labels to utilize samples filtered by the threshold. Both play a positive role in improving pseudo-label confidence, and an appropriate α value maximizes their effects by balancing the weights of the two components, thereby alleviating confirmation bias.

**Figure 5:**
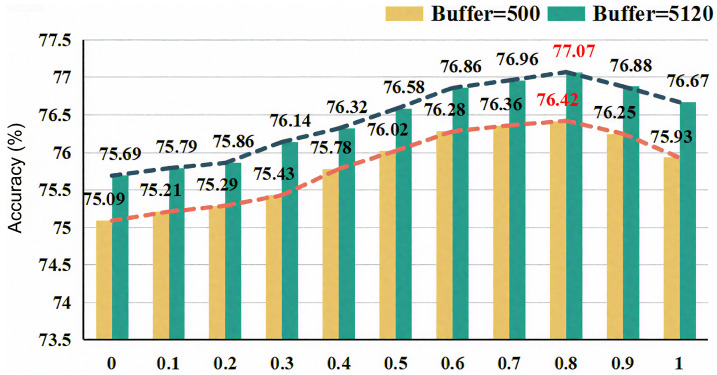
The impact of different parameter *T* on the CIFAR-10 dataset. Yellow and green bars represent results with buffer sizes of 500 and 5120, respectively; the orange and dark-blue dashed lines indicate the corresponding trend curves; red numbers mark the peak performance points.

Figure 5 illustrates the impact of the threshold parameter *T* (in Equation (19)) on model performance. This experiment is also conducted on the CIFAR-10 dataset with a 0.8% labeled data ratio. From the experimental results, we can observe that the influence trend of parameter *T* on model performance is similar, whether the buffer size is 500 or 5120.

The threshold *T* controls the sparsity of edges in the pseudo-label graph: as *T* increases, the connections between samples with low pseudo-label similarity are disconnected, and then the contrast loss pushes these low-similarity samples away from each other in the feature space. An appropriate *T* value can construct a more accurate pseudo-label graph to guide feature learning, thereby alleviating feature bias. However, when T=1, the guided contrastive learning loss is equivalent to a self-supervised loss. At this point, the model treats other samples of the same category as negative examples, leading to learned features that lack discriminability and, in turn, reducing the model’s performance.

## 5. Conclusions

This paper proposes a Bias Calibration (BiCa) method for semi-supervised continual learning in image classification, which mitigates confirmation bias and feature bias through the novel integration of Confidence-Enhanced Learning [8] and Guided Contrastive Learning. Confidence-Enhanced Learning obtains high-confidence pseudo-labels by reducing competition between similar classes and penalizing low-confidence predictions, thereby alleviating confirmation bias. Guided Contrastive Learning constructs pseudo-label graphs and feature representation graphs, and then uses the pseudo-label graph to guide the optimization of the feature representation graph, improving the model’s ability to distinguish between different classes. Extensive experiments verify the effectiveness and generality of BiCa.

Despite the promising results achieved by the proposed BiCa method, there are certain limitations that warrant further investigation. First, our framework heavily relies on an experience replay buffer to mitigate forgetting. As the number of continuous tasks increases, maintaining a representative subset of historical data in a fixed-size buffer can become a bottleneck, especially on edge sensor devices with severe memory constraints. Second, in extreme real-world open environments where sensor data streams might contain severe noise or substantial out-of-distribution (OOD) anomalies, the quality of generated pseudo-labels could degrade, potentially leading to error accumulation.

In future work, we plan to address these limitations from two perspectives. On one hand, we will explore robust OOD detection mechanisms and memory-efficient generative replay strategies to reduce reliance on raw data storage. On the other hand, bridging the gap between foundation models and edge sensors is a promising trajectory. We intend to investigate lightweight continual learning paradigms by integrating structurally learnable adapters and token compression techniques into Vision Language Models (VLMs), thereby unlocking the zero-shot generalization capabilities of large models for resource-constrained sensor platforms without incurring prohibitive computational overhead.

## Figures and Tables

**Table 1:** Average accuracy (%) on the CIFAR-10 dataset.

Method	Buffer Size = 500			Buffer Size = 5120		
	0.8%	5%	25%	0.8%	5%	25%
ER [24]	36.31	51.89	60.92	37.40	64.11	79.72
iCaRL [25]	24.72	35.81	60.93	20.71	35.50	56.32
DER [6]	29.10	35.31	50.03	32.91	47.60	73.92
GDumb [26]	39.61	40.89	44.81	40.80	71.21	81.42
PseudoER [27]	50.51	56.53	57.02	55.41	70.02	71.50
CIC [27]	47.40	61.72	65.21	48.91	72.70	82.81
CCIC [27]	54.01	63.32	63.93	55.20	74.31	84.72
PAWS [28]	51.80	64.61	65.92	53.91	72.74	76.67
NNCLS [7]	73.21	77.20	77.31	73.71	79.32	81.00
NI-SSCL [29]	73.71	78.30	79.53	74.28	80.65	83.23
BiCa (Ours)	76.42	77.98	78.94	77.07	81.79	84.11

**Table 2:** Average accuracy (%) on the CIFAR-100 dataset.

Method	Buffer Size = 500			Buffer Size = 5120		
	0.8%	5%	25%	0.8%	5%	25%
ER [24]	8.21	13.73	17.11	9.60	22.81	37.92
iCaRL [25]	3.61	11.33	27.62	4.31	12.20	30.92
DER [6]	1.71	5.13	13.04	1.61	4.72	11.90
GDumb [26]	8.62	9.93	10.09	9.61	23.30	33.22
PseudoER [27]	8.71	11.43	12.34	15.12	24.91	30.10
CIC [27]	11.13	17.12	20.41	11.80	26.71	41.62
CCIC [27]	11.52	19.51	20.34	12.01	29.52	44.30
PAWS [28]	16.13	21.21	19.20	16.20	32.89	42.39
NNCLS [7]	27.41	31.42	35.31	27.51	46.01	56.41
NI-SSCL [29]	28.28	33.30	40.01	29.87	49.81	57.86
BiCa (Ours)	28.54	32.53	36.62	28.61	48.21	58.73

**Table 3:** Average accuracy (%) on the ImageNet-100 dataset.

Method	1%	5%	25%
ER [24]	12.60	26.65	39.31
FOSTER [30]	15.36	33.51	42.85
X-DER [25]	11.35	28.23	45.82
CCIC [27]	14.13	19.85	26.34
PAWS [28]	27.22	48.09	56.55
NNCLS [7]	29.92	51.35	65.75
BiCa (Ours)	30.93	52.74	67.16

**Table 4:** Ablation experiments of different components on the CIFAR-10 and CIFAR-100 datasets.

Components				CIFAR-10			CIFAR-100		
$L_{\mathrm{NNCSL}}$	$L_{\mathrm{EML}}$	$L_{\mathrm{ANL}}$	$L_{\mathrm{GCL}}$	**0.8%**	**5%**	**25%**	**0.8%**	**5%**	**25%**
✔				73.21	77.20	77.31	27.41	31.42	35.31
✔	✔			73.78	77.42	77.74	27.65	31.62	35.70
✔		✔		73.83	77.33	77.81	27.59	31.66	35.63
✔			✔	75.56	77.73	78.47	28.02	32.04	36.33
✔	✔	✔		74.47	77.52	78.32	27.96	31.89	35.98
✔	✔		✔	75.85	77.84	78.65	28.19	32.27	36.47
✔		✔	✔	75.71	77.78	78.72	28.25	32.21	36.39
✔	✔	✔	✔	**76.42**	**77.98**	**78.94**	**28.54**	**32.53**	**36.62**

*Note:* ✔ indicates the corresponding loss/component is enabled. Bold numbers indicate the best performance in each column.

## Data Availability

The datasets used in this study are publicly available: CIFAR-10 and CIFAR-100 can be accessed from the official CIFAR repository (https://www.cs.toronto.edu/~kriz/cifar.html (accessed on 6 April 2026)); ImageNet-100 is available from the ImageNet dataset portal (https://image-net.org/ (accessed on 6 April 2026)).
